# Study of Healthcare Personnel with Influenza and other Respiratory Viruses in Israel (SHIRI): study protocol

**DOI:** 10.1186/s12879-018-3444-7

**Published:** 2018-11-06

**Authors:** Avital Hirsch, Mark A. Katz, Alon Laufer Peretz, David Greenberg, Rachael Wendlandt, Yonat Shemer Avni, Gabriella Newes-Adeyi, Ilan Gofer, Maya Leventer-Roberts, Nadav Davidovitch, Anat Rosenthal, Rachel Gur-Arie, Tomer Hertz, Aharona Glatman-Freedman, Arnold S. Monto, Eduardo Azziz-Baumgartner, Jill Morris Ferdinands, Emily Toth Martin, Ryan E. Malosh, Joan Manuel Neyra Quijandría, Min Levine, William Campbell, Ran Balicer, Mark G. Thompson, on behalf of the SHIRI workgroup

**Affiliations:** 1 Chief Physician’s Office, Clalit Health Services, Clalit Research Institute, Tel Aviv, Israel; 2 School of Public Health, Medical School for International Health, Faculty of Health Sciences, Ben Gurion University of the Negev, Beer Sheva, Israel; 3 Department of Epidemiology, University of Michigan School of Public Health, Ann Arbor, MI USA; 4 Rabin Medical Center, Occupational Medicine Department, Petah Tikva, Israel; 5 Pediatric Infectious Disease Unit, Soroka University Medical Center, Beer Sheva, Israel; 6 Abt Associates, Inc, Atlanta, GA USA; 7 Clinical Virology, Soroka University Medical Center, Ben Gurion University of the Negev, Beer Sheva, Israel; 8 Department of Health Systems Management, School of Public Health, Faculty of Health Sciences, Ben Gurion University of the Negev, Beer Sheva, Israel; 9 Department of Microbiology Immunology and Genetics, Faculty of Health Sciences, Ben Gurion University of the Negev, Beer Sheva, Israel; 10 Vaccine and Infectious Disease Division, Fred Hutch Cancer Research Center, Seattle, WA USA; 11 Israel Center for Disease Control, Ministry of Health, Tel Hashomer, Ramat Gan, Israel; 12 Influenza Division, Centers for Disease Control and Prevention (CDC), Atlanta, GA USA; 13 U.S. Naval Medical Research Unit N° 6 – Lima, Lima, Peru; 14 Department of Epidemiology and Preventive Medicine, School of Public Health, Sackler Faculty of Medicine, Tel Aviv University, Tel Aviv, Israel

## Abstract

**Background:**

The Study of Healthcare Personnel with Influenza and other Respiratory Viruses in Israel (SHIRI) prospectively follows a cohort of healthcare personnel (HCP) in two hospitals in Israel. SHIRI will describe the frequency of influenza virus infections among HCP, identify predictors of vaccine acceptance, examine how repeated influenza vaccination may modify immunogenicity, and evaluate influenza vaccine effectiveness in preventing influenza illness and missed work.

**Methods:**

Cohort enrollment began in October, 2016; a second year of the study and a second wave of cohort enrollment began in June 2017. The study will run for at least 3 years and will follow approximately 2000 HCP (who are both employees and members of Clalit Health Services [CHS]) with routine direct patient contact. Eligible HCP are recruited using a stratified sampling strategy. After informed consent, participants complete a brief enrollment survey with questions about occupational responsibilities and knowledge, attitudes, and practices about influenza vaccines. Blood samples are collected at enrollment and at the end of influenza season; HCP who choose to be vaccinated contribute additional blood one month after vaccination. During the influenza season, participants receive twice-weekly short message service (SMS) messages asking them if they have acute respiratory illness or febrile illness (ARFI) symptoms. Ill participants receive follow-up SMS messages to confirm illness symptoms and duration and are asked to self-collect a nasal swab. Information on socio-economic characteristics, current and past medical conditions, medical care utilization and vaccination history is extracted from the CHS database. Information about missed work due to illness is obtained by self-report and from employee records. Respiratory specimens from self-collected nasal swabs are tested for influenza A and B viruses, respiratory syncytial virus, human metapneumovirus, and coronaviruses using validated multiplex quantitative real-time reverse transcription polymerase chain reaction assays. The hemagglutination inhibition assay will be used to detect the presence of neutralizing influenza antibodies in serum.

**Discussion:**

SHIRI will expand our knowledge of the burden of respiratory viral infections among HCP and the effectiveness of current and repeated annual influenza vaccination in preventing influenza illness, medical utilization, and missed workdays among HCP who are in direct contact with patients.

**Trial registration:**

NCT03331991. Registered on November 6, 2017.

**Electronic supplementary material:**

The online version of this article (10.1186/s12879-018-3444-7) contains supplementary material, which is available to authorized users.

## Background

Healthcare personnel (HCP) are believed to be at increased risk of respiratory viral infections, with one in five estimated to be infected with seasonal influenza each year [1]. These estimates vary widely, however, depending on the extent of active surveillance and the use of serologic vs. molecular diagnostics. Respiratory infections are of particular concern among HCP because of the close contact of HCP with patients [2], and the risk of HCP transmitting respiratory viruses to others [3]. Although recent research suggests that certain subgroups of HCP, such as those who perform aerosol-generating procedures, may be at heightened risk of infection with influenza and other respiratory pathogens [4], incidence of infections among HCP is not well characterized across different occupations and responsibilities. Furthermore, although HCP often work while ill [5–7], the extent to which infected HCP transmit respiratory pathogens to patients is not clear [8]. Further research is needed on the frequency and types of interactions HCP have with patients when they have symptomatic, atypical, or asymptomatic [1, 9] influenza virus infections.

Vaccination of HCP against influenza is an important component of infection control in healthcare settings [10], but persistently low rates of vaccine uptake among HCP in most countries remains an international concern [10–12]. Numerous studies on the knowledge, attitudes, and practices (KAP) associated with influenza vaccine acceptance and rejection have been conducted among HCP in high-income countries in North America and Europe [13–16]. However, much less is known about the barriers to vaccine acceptance among HCP in countries in the Middle East [12]. In Israel, a high-income country, less than half (49%) of HCP received influenza vaccine for the 2015–2016 influenza season; vaccine coverage was somewhat higher among hospital-based physicians (53%) than nurses (46%) [17]. More information is needed on the occupational, socio-demographic, and KAP factors that may explain variations in influenza vaccine acceptance in Israel, including how the personal KAP of HCP may impact on their promotion of influenza vaccination among their patients.

Although recent reviews confirm that the seasonal influenza vaccine is moderately effective in reducing the risk of influenza illness among adults [18], multiple gaps in knowledge remain about the preventive value of the vaccine among HCP [1]. To date, there has been only one randomized controlled trial of influenza vaccine efficacy among HCP, and this study measured serologic outcomes only [19]. Given that vaccinated individuals are less likely to seroconvert after an influenza virus infection and so can appear as not having been infected [20, 21], the use of serologic outcomes likely biases (and specifically, inflates) vaccine efficacy estimates.

Gaps are especially evident in our understanding of the value of influenza vaccine for reducing secondary adverse outcomes, including nosocomial infections among patients [22, 23] and missed work due to illness [24, 25]. Evidence is also needed to validate the potential of the vaccine in reducing the severity and duration of disease and diminish infectiousness among those who have breakthrough infections despite being vaccinated [24, 26]. Because HCP often receive influenza vaccine during multiple years, studies of HCP also provide an opportunity to examine how prior vaccinations may modify immunogenicity and vaccine effectiveness, and may provide answers to questions regarding the extent of residual protection and/or negative vaccine interference across seasons [27–29].

Here we provide an overview of the design and methods of a prospective study of influenza vaccine effectiveness in HCP named “Study of Healthcare Personnel with Influenza and other Respiratory Viruses in Israel” (SHIRI). The SHIRI cohort will follow approximately 600 HCP, all of whom have direct patient contact, during at least three influenza seasons, and an additional 1400 HCP during at least two influenza seasons.

Our study has four primary objectives: (1) to describe the frequency of respiratory, atypical (e.g., febrile only), and asymptomatic influenza virus infection among HCP; (2) to identify predictors of vaccine acceptance (and hesitancy) among HCP; (3) to examine how repeated influenza vaccination may modify immunogenicity; and (4) to evaluate influenza vaccine effectiveness in preventing influenza illness, associated missed work, and working while ill. Table 1 lists the knowledge gaps we identified within each of these aims and the study features intended to address these gaps.

**Table 1:** Study Goals and Features Intended to Address Specific Knowledge Gaps

Knowledge Gap	Study Feature
1. Description of the frequency of influenza virus infection among healthcare personnel, including those manifesting as acute respiratory illness, atypical illness, or asymptomatic infection	
Studies of influenza illness among HCP using laboratory-confirmed outcomes are scarce.	Identification of symptomatic influenza virus infections with mqRT-PCR assay.
Typical surveillance strategies have focused on acute respiratory illness using highly specific case definitions which overlook non-respiratory and non-febrile manifestations of influenza disease.	Usage of a broad case definition: “illness with cough, runny nose, body aches, or feverishness in the past seven days.”
Few studies have used both molecular and serologic diagnostics to assess the total burden of influenza virus infection among HCP.	In addition to mqRT-PCR, 4-fold increases in HI from pre- to post-season will also be used to identify possible influenza virus infection among unvaccinated HCP.
It is unclear how differences in sex, age, occupation, and underlying health may contribute to the frequency of influenza illness among HCP.	Usage of random stratified sampling to enroll a mixture of HCP by sex, age, and occupation. Assess underlying health status by self-report and medical record extraction.
Further research is needed on whether specific HCP roles and responsibilities increase the risk of infection with influenza and other respiratory pathogens.	Comparison of the frequency of ARFI (and infection with influenza and other respiratory viruses) by number of hours of direct patient care and by performance of aerosol-generating procedures (such as suction of fluids and tracheal intubation).
More information is needed on the impact of influenza illness on HCP’s absence from work due to illness and working while ill.	Assessment of the duration of illness, missed, and rescheduled work due to illness, hours worked during illness, and ability to do usual activities.
2. Identification of predictors of vaccine acceptance (and hesitancy)	
Most studies of HCP have focused on influenza vaccine uptake in specific seasons and less on behavior over multiple years.	Description of how the frequency of influenza vaccination during the five years prior to enrollment and during the two to three years of participation in the cohort varies by sex, age, occupation, and socio-economic status.
Most studies of KAP associated with influenza vaccination among HCP have been conducted in the United States or Western European countries.	This study is conducted in Israel, and will examine KAP topics including association between frequency of vaccination and perceived susceptibility to influenza, perceived benefits and risks of influenza vaccination, readiness to be vaccinated, and anticipated worry and regret about influenza vaccination decisions.
3. Examination of how repeated influenza vaccination may modify immunogenicity	
Few studies have assessed the effects of repeated influenza vaccination across multiple seasons on immunogenicity.	Examination of how HI differs depending on the receipt of influenza vaccines up to ten years prior to the study for consistent health plans members.
Further research is needed on the mechanisms through which prior vaccination affects immunogenicity.	Examination of whether any link between repeated vaccination and HI can be explained by HCP’s “antibody landscape”.
Further research is needed on whether repeated prior vaccination impacts cell mediated immune response to influenza vaccines.	In a subset of participants who provide peripheral blood mononuclear cells before and after vaccination, examination of whether repeated prior vaccination is associated with suppression of B-cell and T-cell immunogenicity.
4. Evaluation of influenza vaccine effectiveness in preventing influenza illness and associated missed work and working while ill	
Prior study of IVE among HCP used serologic outcomes, which are likely biased among vaccinees.	Estimate the effectiveness of the influenza vaccine in preventing mqRT-PCR confirmed influenza illness among HCP.
It is unclear whether influenza vaccines may reduce missed work due to influenza illness or reduce time spent working while ill (i.e., presenteeism) with influenza.	Examine the hours of missed work and presenteeism between the dates of onset and resolution of influenza illness; apply these observations to estimate potential IVE in averting missed work or presenteeism.
More information is needed on the extent to which prior vaccination may offer residual protection and/or interfere with IVE in subsequent seasons.	Examination of IVE associated with combinations of current season vaccination and frequent vs. infrequent prior vaccinations.
Further research is needed on whether the influenza vaccination may modify influenza disease severity and duration among those who become infected despite vaccination.	Among HCP with influenza illness, examination of whether symptom severity and illness duration are lower among vaccinated vs. unvaccinated HCP.

Abbreviations: *HCP* healthcare personnel, *mqRT-PCR* multiplex quantitative real-time reverse transcription polymerase chain reaction, *HI* hemagglutination inhibition, *ARFI* acute respiratory illness or febrile illness, *KAP* knowledge, attitudes, and practices, *IVE* influenza vaccine effectiveness

The findings of this study will contribute to our knowledge about the burden of influenza and the vaccine effectiveness among HCP, and thus may influence a vaccination policy change in Israel and internationally.

## Methods/design

### Study design

**Fig. 1:**
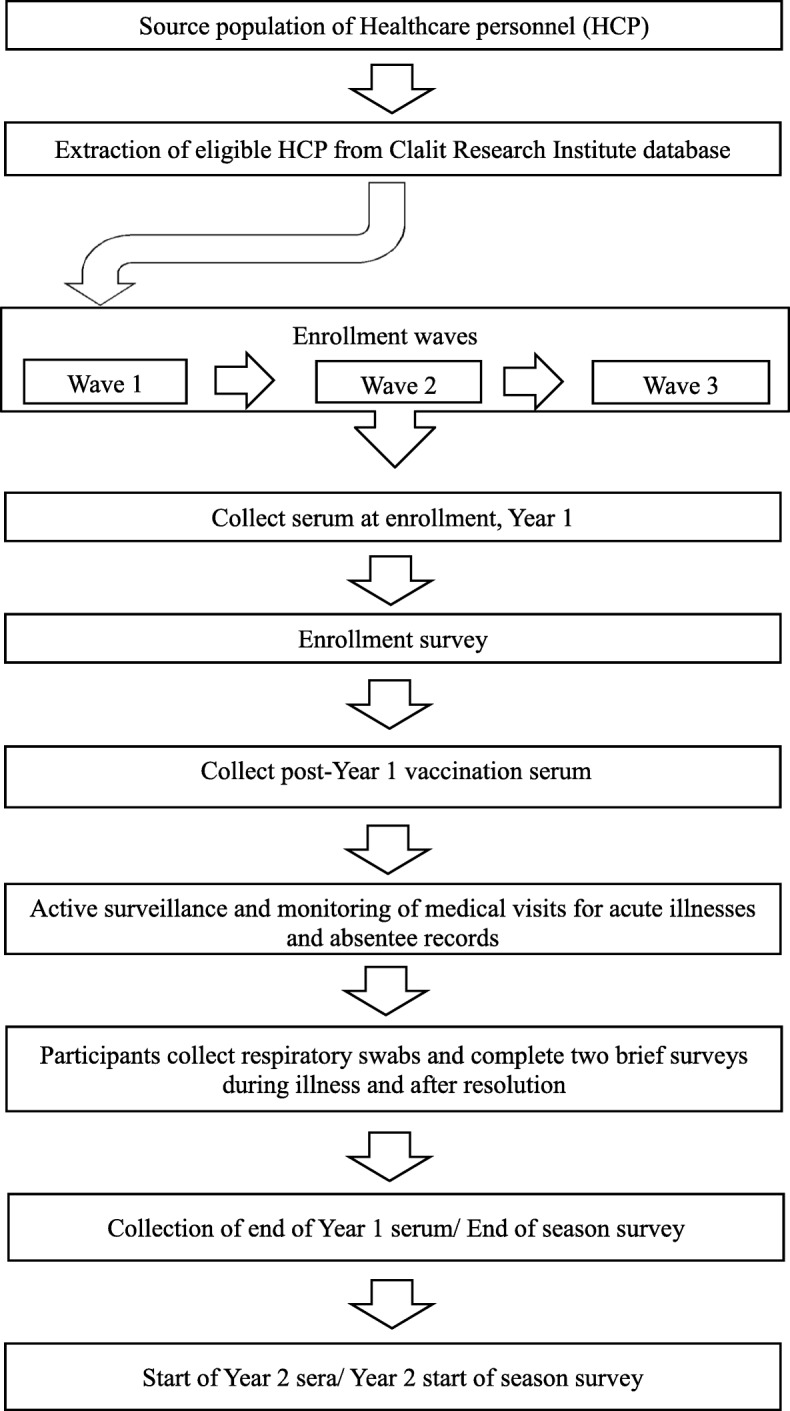
Steps in Recruitment, Enrollment, and Follow-up

This prospective cohort study of influenza vaccine effectiveness in HCP is funded by the United States Centers for Disease Control and Prevention (US CDC). A steering committee, consisting of principal investigators from the two hospitals, Clalit Health Services (CHS), the Israeli Center for Disease Control, Abt Associates, and the US CDC was established and charged with making critical decisions related to the study. Additional investigators from the University of Michigan School of Public Health and the Ben Gurion University School of Public Health will advise on laboratory methods and qualitative methods related to the study. Study activities are described in Fig. 1.

### Setting

The study is being conducted among HCP at two hospitals in Israel: (1) Soroka University Medical Center (located in Beer Sheva, a city in southern Israel), and (2) Beilinson Hospital (located in Petah Tivka, a city in central Israel) (Table 2). Both hospitals are managed and primarily staffed by CHS, the largest insurer and integrated care provider in Israel with over 4.4 million members in 2017, which constitute over 50% of the population.

**Table 2:** Study Sites

Name of study site	Location	Number of employees	Number Of beds	Number of eligible participants, 2016^c^	Number of eligible participants, 2017^d^
Soroka University Medical Center	Beer Sheva, Israel	4300^b^	1074^a^	2436	2853
Beilinson Hospital	Petah Tikva, Israel	5500^b^	850^b^	1988	2260

^a^Data as of 2016; Data were extracted from Israeli Ministry of Health reports

^b^Data of 2017; Data were extracted from employee records from the CHS EHR and Israeli Ministry of Health reports

^c^Recruitment in 2016 began in October, when nationwide vaccination was already in progress. HCP who had already been vaccinated in the current season were not eligible for the study

^d^Excludes participants enrolled in 2016

Number of beds data was obtained from Israeli Ministry of Health reports

Abbreviations: *CHS* Clalit Health Services, *EMR* electronic medical record, *HCP* healthcare personnel

### Participants

#### Eligibility criteria

Eligible participants include HCP employed at a participating hospital who meet the following criteria: at least 18 years old; work full-time (≥30 h per week); and have routine direct hands-on or face-to-face contact with patients (within one meter) as part of a typical work shift. These eligibility criteria are similar to previous studies of HCP with direct patient contact [24, 30]. Participation is offered to physicians, nurses, respiratory therapists, physical therapists, unit clerks, radiograph technicians, medical assistants, transporters, and other HCP who have direct contact with patients. All participants must also be members of CHS and have had continuous membership in CHS for at least 1 year prior to enrollment (in order to access medical and vaccination records). HCP are invited to join the study whether or not they intend to be vaccinated. HCP are excluded if they received the current season’s flu vaccine more than 48 h before enrollment. If enrollment goals are not met, some enrollment restrictions can be eliminated (e.g., requiring prior year membership in the Clalit health plan, working, ≥30 h per week, or plans to stay at the facility for at least 2 years) and differences in outcomes by these characteristics can be examined analytically.

Eligibility is determined by reviewing CHS’s electronic medical record (EMR), which contains information on all of the eligibility criteria listed above except for the number of working hours, which is determined in the recruitment phone call. EMR data is extracted at the Clalit Research Institute, the research arm of CHS. Screening information is logged in the Research Electronic Data Capture (REDCap), a common database (described below).

#### Recruitment

In order to recruit HCP with diverse socio-demographic characteristics, occupational responsibilities, and influenza vaccination histories, eligible HCP were invited to participate using a stratified sampling strategy that targeted eligible participants in groups (strata) categorized by sex, age, occupation, and previous year influenza vaccination status. This systematic approach was intended to minimize convenience sampling, which can introduce both known and unknown biases. Each group includes individuals with unique characteristics known to potentially affect influenza vaccine immunogenicity and effectiveness.

At CHS, a list of eligible HCP at each facility was generated and categorized into groups based on age (18–34, 35–49, ≥50 years old), sex, occupational categories (1- Physicians; 2 - Nurses, medical therapists, and professional technicians; and 3 - Medical assistants and support staff), and prior season influenza vaccination status (yes vs. no). Thus, prior to each year of the study, the sample is drawn from 36 unique strata (2 sexes * 3 age groups * 3 occupation groups * 2 prior season vaccination status groups). This stratified sampling strategy will be applied to potential new participants each year.

At each hospital, goals are set for the minimum and maximum number of enrollees per strata; then, recruitment is implemented in three waves. During the first wave of recruitment, HCP from the 36 strata are invited at random to participate in the study. Next, in Wave 2, we identify study strata where additional recruitment is needed and expand direct invitations to additional HCP who have not yet been invited to join the study. In strata where all HCP have been contacted but recruitment goals have not been met, we will recruit similar HCP, prioritizing HCP who are similar in previous vaccination status, followed by age, sex, and lastly by profession. Finally, in Wave 3, we accept volunteers to meet the total sample goals for each facility. All potential enrollees will be recorded in a recruitment log in order to track invitations, acceptance, and refusal and reasons for refusal.

#### Timeline of enrollment

A total cohort of 2000 HCP will be enrolled over a three-year period. Enrollment periods are targeted prior to influenza seasons. During Year 1 of the study, which targeted the 2016/2017 influenza season, enrollment could not begin until October 6, 2016 due to funding and institutional approval delays. Recruitment continued until the start of the influenza season (December 4, 2016) for a total of 8 weeks. This recruitment period included a month of frequent Israeli national holidays (October 2016), during which enrollment was challenging because many HCP were on vacation. In Year 1 of the study, 596 HCP were enrolled, and will contribute to Years 1, 2 and 3 of this 3-year cohort study. Enrollment in Year 2 began in June 2017. New enrollees in Year 2 will contribute to Years 2 and 3 of the study.

### Active surveillance

**Fig. 2:**
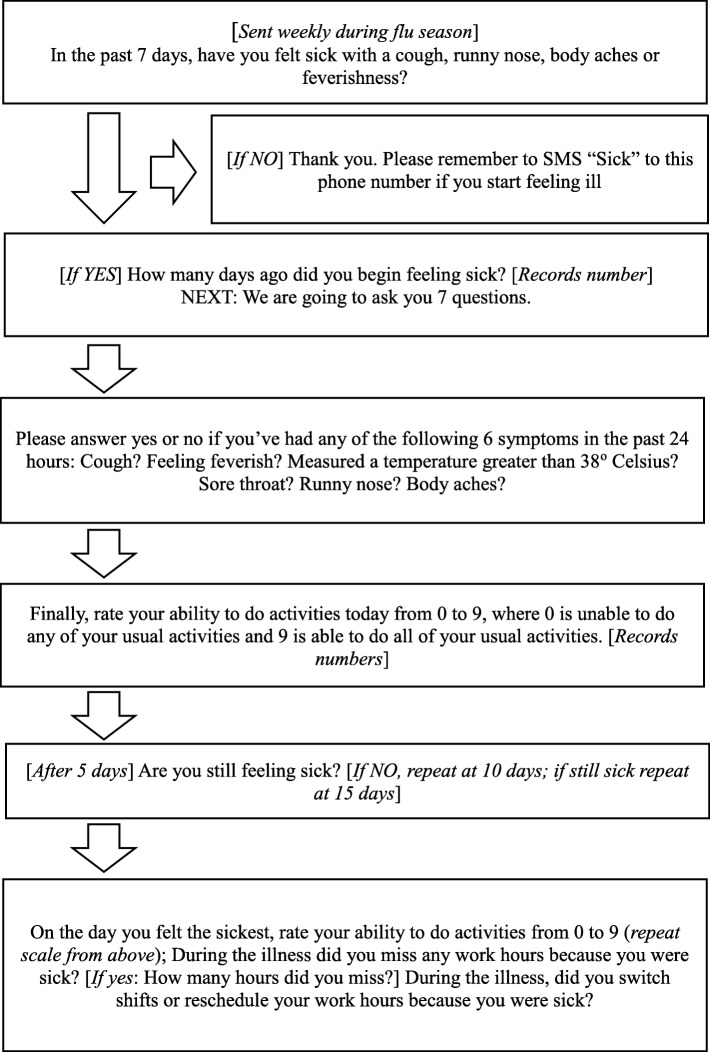
Active Surveillance SMS Messaging Flow

During the influenza season, participants receive twice-weekly short message service (SMS) messages asking them to confirm whether they have acute illness symptoms, defined as one or more of the following symptoms within the past 7 days: cough, runny nose, body aches, or feverishness. In addition to these SMS messages, participants are asked to contact the study staff immediately when they experience symptoms of acute illness. If a participant reports an acute illness, he or she is asked follow-up questions about specific symptoms and date of onset. Participants are first contacted by SMS message. If they do not respond, they are contacted by telephone. If participants do not respond to phone calls, an attempt is made to contact them in-person. Ill participants receive follow-up SMS messages about whether the illness has resolved. Once an illness resolution is reported by a participant, he or she is sent five follow-up questions by SMS message about illness presentation, duration, and impact on work attendance. These surveillance activities are described in Fig. 2.

The start and end of active surveillance is determined by the study investigators and steering committee based on historical patterns for seasonal influenza circulation and available clinical and surveillance indicators of laboratory-confirmed influenza virus circulation in Israel. In the first year of the study, active surveillance started on December 4, 2016 and continued through March 23, 2017.

### Data collection

We collect data for all participants from multiple sources. Key variables and their sources in the years prior to the study and during each study year are summarized in Table 3.

**Table 3:** Key Variables and Sources of Information for Participants

	Self-reported		Electronic Medical Records		Time Period	
	Enrollment survey	EOS survey	EMR	Employee Records	From year(s) prior to the study	Data from years enrolled in study
Demographic						
Sex					✓	
Date of birth					✓	
Marital status	✓		✓			✓
Country of birth			✓		✓	
Immigration date			✓		✓	
Ethnicity by country of birth of individual, the parents, or grandparents			✓		✓	
Socio-economic status by clinic address			✓		✓	✓
Supplementary insurance status			✓		✓	
Dates enrolled as CHS member			✓		✓	
Level of education	✓					✓
Household composition (number of rooms; number of family members in the house)	✓					✓
Occupation and work responsibilities	✓				✓	✓
Family income	✓					✓
Health Status and Risk Behaviors						
Health status and health behaviors	✓	✓			✓	
Smoking status, history			✓		✓	
Pack years			✓		✓	
Height			✓		✓	
Weight			✓		✓	
Body mass index			✓		✓	
Medication use for chronic conditions and immunosuppressants			✓		✓	
Attitudes						
Perceptions of illness, vaccines, missing work	✓					✓
Recollection of influenza vaccination (for vaccinated HCP)		✓				✓
Reasons for not receiving the influenza vaccine (for unvaccinated HCP)		✓				✓
Job satisfaction	✓					✓
Influenza Vaccination Documentation						
Vaccine administration date			✓		✓	✓
Vaccine type			✓		✓	✓
Vaccine manufacturer & lot			✓		✓	✓
Employee Records of Illness Absences				✓		✓
Acute Respiratory Illness						
Number of inpatient admissions associated with acute illness			✓		✓	✓
Chronic Medical Conditions and Pregnancy						
Number of ambulatory or inpatient medical encounters associated with chronic medical condition			✓		✓	✓
Chronic medical conditions			✓		✓	
Pregnancy					✓	✓

Abbreviations: *EOS* end of season, *EMR* electronic medical record, *CHS* Clalit health plan, *HCP* healthcare personnel

#### Electronic medical records

Information on socio-demographic characteristics, current medical conditions, medical history, medical care utilization, and influenza vaccination history are extracted from the CHS EMR at enrollment and at the end of each study year. For all participants, chronic medical conditions are identified using a combination of the International Classification of Diseases, Ninth Revision (ICD-9) codes from inpatient and outpatient records, International Classification of Primary Care Coding (ICPC), physician-entered free text diagnoses from outpatient medical records, and electronic chronic disease registries maintained by the Clalit Research Institute (details in Additional file 1: Annex 1 and Annex 2).

Routine influenza vaccine administration for CHS HCP members who receive the vaccine is recorded in the health fund’s EMR. Because the vaccine is offered free of charge for members in CHS hospitals and clinics, nearly all of those who are vaccinated do so in such settings. Participants are considered vaccinated for a specific season if they received influenza vaccine from September 1 through March 31 of the relevant influenza season. To date, influenza vaccine has not been offered in Israel prior to September 1. We document prior vaccine history, according to EMR records, from the the 2006–2007 season, 10 years prior to the first year of our study.

In addition, we document medical visits for ARFI during weeks of active surveillance in every study year. We also document medically attended ARFI in the 10 years prior to enrollment, distinguishing between medical visits that occurred during the influenza season and those that occurred outside of the influenza season. Medically attended ARFI episodes during active surveillance and for years prior to enrollment are captured through evaluation of outpatient visits, emergency room visits, and hospital admissions. Criteria used for medically attended ARFI are described in Additional file 1: Annex 3.

Outpatient visits, emergency room visits, medications dispensed at CHS pharmacies, and hospital admissions are regularly updated in the CHS EMR, making it possible to document medical visits nearly in real-time by recording ICD-9 codes and free text entered by physicians. We also access the EMR to document influenza antiviral medications and antibiotics dispensed during acute illnesses.

#### Self-reported data

At enrollment and at the beginning of each subsequent season, all participants complete a brief survey with questions about socio-demographic characteristics that are not available in the EMR, occupational responsibilities, health status, and KAP regarding seasonal influenza vaccination.

At the end of each influenza season, participants are asked to complete another brief survey that includes questions about their overall health and to describe previously unreported illnesses (‘End-of-season survey’). Surveys are designed to be self-administered electronically through the Internet using the REDCap system.

#### Work absenteeism

During active surveillance, we document days of missed work associated with respiratory illnesses. Missed workdays are identified by self-report (as part of acute illness and illness follow-up SMS messages) and by periodic reviews of human resource department employee absentee records. Illness days are identified as all days between self-reported illness onset and illness resolution dates (from SMS surveillance messages or by direct phone screening). Thus, missed work due to illness includes days that participants directly reported missing due to illness and days of absence (according to employee records) between the illness onset and resolution dates.

### Laboratory methods

#### Nasal specimens

When participating HCP report being ill with a respiratory illness during the influenza season, they are instructed to self-collect a nasal swab using a self-swabbing kit [31] that includes illustrated instructions, a nasal mid-turbinate swab, and a tube with room temperature transport medium. Respiratory specimens are placed in viral transport medium and delivered to the respective hospitals from HCP’s residences by a courier service or are hand-carried by participants to study staff at their respective hospitals. If participants prefer not to collect the nasal swab themselves, study staff are able to collect it for them.

#### Molecular diagnostics

Specimens from Beilinson Hospital are stored at 4 °C and delivered by courier to the Clinical Virology Laboratory (CVL) at Soroka University Medical Center twice a week, where they are immediately aliquoted and frozen at − 80 °C. Specimens from participating Soroka University Medical Center HCP are brought to the laboratory daily, where they are aliquoted and frozen.

After one freeze-thaw cycle, specimens are tested for influenza A viruses [A(H1N1)pdm09 and A(H3N2)], influenza B viruses, respiratory syncytial virus (RSV), human metapneumovirus, and coronaviruses (NL63, 229E, OC43, HKU1) using validated multiplex quantitative real-time reverse transcription polymerase chain reaction (mqRT-PCR) assays [32], with protocols, primers, probes, and reagents supplied by Hy-labs (Israel) and Integrated DNA Technologies (USA). The CVL, in collaboration with the University of Michigan (Ann Arbor, Michigan, US), has completed the World Health Organization and US CDC influenza proficiency panels. Quality Control for Molecular Diagnostics (Glasgow, Scotland, UK) proficiency panels were also completed for influenza, RSV, human metapneumovirus (hMPV) and coronaviruses.

Some specimens will have molecular characterization with genetic sequencing and other assays to detect genetic markers to determine viral subclades and antiviral resistance at a reference laboratory approved by the study steering committee. Remaining aliquots of all study specimens may be sent to a US CDC-designated facility (also approved by the study steering committee) for additional virus characterization, banking, and storage. No specimens will contain personal identifiers.

##### Blood Specimens

At enrollment, prior to each influenza season, 10 mL specimens of whole blood are collected from all participants. In addition, 5 mL specimens of whole blood are collected at the end of season 1 and the start and end of subsequent seasons. Participants who received the influenza vaccine during the study period will be asked to provide an additional sample of 5 mL of whole blood approximately 28 days (within a range of 21–42 days) after vaccination. Sera are extracted from whole blood and stored frozen until testing.

Participants who consent to providing additional blood at enrollment, as an optional part of the study, provide an additional 10 mL heparinized whole blood at enrollment and the end of season for extraction of peripheral blood mononuclear cells (PBMC). In addition, we collect 10 mL of heparinized whole blood approximately 7 days after vaccination from participants who agree to this optional part of the study. All PBMC samples are centrifuged, undergo cell count, diluted to 1-5 × 10^6^ cells/ml and gradually frozen to − 80 °C, and placed in liquid nitrogen within 24 h. PBMC samples will be used for cell-mediated immunity assays.

#### Hemagglutination inhibition assay

The hemagglutination inhibition (HI) assay will be used to detect the presence of influenza antibodies in serum. HI to inactivated influenza vaccine components and influenza circulating strains will be performed at a reference laboratory using standard methods [33] as described previously [28, 34] (See Additional file 1: Annex 4). Egg-grown viruses will be supplied by the US CDC’s International Reagent Resource. Preparation of serum samples will include (a) treatment with a receptor-destroying enzyme to remove nonspecific inhibitors, and (b) removal of nonspecific agglutinins by serum adsorption with packed red blood cells (RBC). Standard 0.5% turkey RBC will be prepared for influenza A (H1N1)pdm09 antigens and ether-treated B influenza antigens. Given indications that neuraminidase of circulating antigenic clusters of influenza A(H3N2) viruses (since 2014) have acquired the ability to bind to RBC, modified HI assays will be conducted for influenza A(H3N2) antigens using guinea pig red blood cells in the presence of the antiviral oseltamivir carboxylate, which inhibits influenza neuraminidase. Serum will be diluted 2-fold starting from 1:10. The HI titer will be the reciprocal of the serum dilution in the last well with complete HI. The geometric mean titer from duplicate results will be reported; HI < 10 will be considered 5 for the purposes of statistical analyses.

#### Additional immunological assays

Additional serologic testing may occur, including neuraminidase inhibition assay testing, antigen microarray testing [35] and other approaches. Neuraminidase-specific antibodies have been shown to play a role in protection against influenza infection [36, 37]. However, many questions remain about the role of neuraminidase in vaccine effectiveness [38].

### Attitudes toward morbidity due to influenza and other respiratory illnesses

As a sub-study within this project, approximately 15–25 in-depth open-ended qualitative interviews are conducted each year with participants who recently had a wintertime respiratory illness and agree to participate in this sub-study.

Participants are contacted by phone approximately 1 week after confirmation of respiratory illness resolution and asked to participate in an interview. Interviews cover topics including illness experience, including symptoms, duration, perceived severity, and disruption to their daily activities and responsibilities, perceptions of how the illness impacted work responsibilities, perceptions of how the illness impacted life outside of work, and reasons for choosing to receive or not receive the influenza vaccine this season (see Additional file 1: Annex 5). Interviews will be recorded by audio recorder and later transcribed. After transcription, the data are translated from Hebrew to English and qualitatively analyzed using NVivo Software (QSR International, Melbourne, Australia) to identify themed code-words that may point to common patterns of comments across the interviews.

### Data management

#### REDCap

Data collection and site-level management are conducted using REDCap, a browser-based metadata-driven software system (Vanderbilt University, Nashville, TN, USA) [39]. Most study instruments, including the recruitment log, online surveys, and laboratory results, allow for real-time data entry directly into REDCap. Surveys are designed to be self-administered electronically through the Internet using home computers, personal mobile telephones, or on computers or tablets provided at the workplace by study staff. In addition, study staff enter participants’ responses directly into the REDCap database for interviews administered by telephone. Routine quality assurance monitoring is conducted locally by the project manager and centrally by the data coordinator (Abt Associates). Missing or unclear information is corrected through follow-up contact with participants.

#### TextIt

TextIt (TextIt, Kigali, Rwanda) is an online platform that uses logic flows to send tailored SMS messages to participants. Custom decision trees were developed in order to trigger exchanges with participants during active surveillance (described above). Specifically, TextIt documents answers to weekly illness inquiries, dates of illness onset and resolution, ratings of symptoms, and missed work. Study staff perform bi-weekly exports of TextIt data and import the data into an Excel macro-enabled workbook in order to track participant responses and illness events over time (see Additional file 1: Annex 6 for more details about TextIt).

### Statistical considerations

#### Cohort size

The required number of participants depends on multiple factors, including expected influenza attack rate, vaccination coverage, and anticipated study attrition. We estimated that with 2340 participants contributing 5 months at-risk for influenza infection per season, 40% vaccine coverage, and 10% influenza illness attack rate, we would be powered to estimate a true vaccine effectiveness of 50% with confidence intervals that do not overlap with zero.

#### Sample size for incidence calculations

The full cohort provides the sample needs for estimating the frequency (or incidence within the study sample) of influenza illness. Given the larger sample demands for the vaccine effectiveness objectives, we expect to have ample statistical power for most objectives that involve estimating incidence or frequencies.

#### Sample size for immune response to vaccine and infection objectives

To assess the immune response to the vaccine, we plan to collect serum from the cohort members prior to vaccine availability, after vaccination, and at the end of each season. Our study design mirrors approaches used in previous studies that assessed the HI antibody titers among HCP analyzed sera from subgroups of 300–800 participants who provided sera at the same three time points [28]. See Additional file 1: Annex 7 for more details.

#### Data analysis of VE

Rates of acute illness associated with mqRT-PCR confirmed influenza virus infection (influenza illness) will be calculated as the number of influenza illnesses divided by person-time measured in weeks of active surveillance. Regression models will be used to estimate influenza vaccine effectiveness (1 – rate of influenza illness among vaccinated HCP/rate among unvaccinated HCP * 100) with 95% confidence intervals. Adjusted models of influenza vaccine effectiveness will include study year, calendar time (i.e., weeks between illness onset and week of peak of influenza season), and a propensity for vaccination score calculated using multivariable logistic regression [40]. Other potential confounders, including study site, will be examined and included in the adjusted model if they change IVE point estimates by > 5%.

#### Data analysis of immunogenicity

Since distributions of HI titer data are typically highly left-skewed, all statistical analyses will be conducted using log base-2 transformed titer data; results are then back-transformed to the original scale for ease of interpretation [28, 41]. Pre- and post-vaccine draws are assumed to be correlated within each person, thus repeated measures linear mixed models will be fitted to estimate geometric mean titers (GMT) and geometric mean ratios (GMR). Compound symmetric covariance error structures will be assumed for repeated measures within individuals. GMT will be calculated by back-transforming the least squares mean estimates of logged titer data. GMR will be calculated by back-transforming the difference of least squares means of post-vaccination and pre-vaccination logged titer estimates. GMR will be interpreted as the geometric mean fold ratio of post-vaccination titer to pre-vaccination titer. Multivariate estimates adjusted a priori for age and sex. Linear, quadractic, and cubic terms for age will be examined to consider possible nonlinear associations with age. Other covariates (e.g., education, household size, working in a hospital setting) may also be adjusted within multivariable models if they were associated with the number of prior vaccinations and either preseason GMT or post-vaccination GMR among vaccines.

### Ethical considerations

The study protocol and procedures have been reviewed and approved by the Helsinki committees (institutional review board) at both of the study sites, and by Abt Associates (the coordinating institution on which US CDC relies). In addition, extraction of data from the CHS EMR was approved by the Data Use Committee of the Clalit Research Institute.

All participants complete written informed consent in Hebrew. Small gifts (such as a gift card) are given to participants at study milestones like completion of enrollment, blood draws, and completion of end-of-season survey. Influenza test results are given to those participants who ask to receive them. Given the research nature of the laboratory methods and time delays in batch testing, mqRT-PCR findings are not available to inform clinical decisions.

## Discussion

We described the recruitment, enrollment, active surveillance, data collection, laboratory methods, and data management procedures for the SHIRI cohort – a US CDC-sponsored prospective study, overseen by CHS, of approximately 2300 HCP at two hospitals in Israel. In this study, we will describe the frequency of influenza virus infections among HCP in Israel. Using a broad case definition of respiratory illness, we aim to identify a wide range of ARFI in order to assess more accurately the burden of respiratory illness among HCP. Due to CHS’s extensive EMR, we will have the ability to look at HCP influenza vaccination records up to 10 years prior to study enrollment in order to better understand how HI antibody titers differ depending on the number and specific combinations of prior influenza vaccinations. We will also be able to evaluate influenza vaccine effectiveness in preventing medically attended and non-medically attended influenza illness and associated missed work days.

The findings from the SHIRI cohort will increase our understanding of the burden of acute respiratory illnesses, and influenza virus illness specifically, among HCP in Israel. The unique design of the study, which includes comprehensive medical and vaccination data, active surveillance using a broad case definition, and both molecular and serologic diagnostics, will provide important data on influenza vaccine immunogenicity and effectiveness among HCP. The combination of insights about influenza burden and vaccine effectiveness may potentially inform influenza vaccine policy for HCP in Israel and internationally.

### Strengths

Our study benefits from the fact that all enrolled HCP are members of CHS, and therefore extensive information is recorded in the EMR, which provides real-time data about ambulatory visits, hospitalizations, and medication use. In addition, the EMR provides reliable historic data about the participants, such as prior vaccination information, socio-economic variables, and data about health status and health behaviors. In our study, even if an ARFI is not reported through the SMS surveillance system, daily monitoring of the EMR for medical visits and medications will allow us to identify illnesses and contact participants directly in order to collect respiratory specimens and complete the illness survey. Combining our study with EMR data and data from the two hospitals’ human resources departments will also allow us to reliably map the impact of illness on missed work and to evaluate days worked when ill (presenteeism).

In addition, the broad case definition of an ARFI promises to be more sensitive for influenza illness than case definitions used in previous HCP research [24] and should allow us to characterize a continuum of mild to moderately severe illnesses. Another strength is that the two study sites (Soroka University Medical Center and Beilinson Hospital) are among the largest hospitals in Israel, and the populations they serve and the HCP they employ vary in socio-demographic and economic characteristics. Soroka University Medical Center, the only general hospital in the Negev region in southern Israel, serves two main local populations: Jews, who mostly live in urban settings, and Bedouin Arabs, who live in a range of settlement types, from cities to unrecognized rural villages that lack electricity and running water [42, 43]. Beilinson Hospital, located in central Israel, serves a mostly urban population of a relatively higher socio-economic status compared to Soroka University Medical Center. Conducting our study in these two different settings may allow us to identify socio-demographic differences in KAP, vaccine uptake, and influenza illness attack rates. The use of mixed methods, including laboratory, clinical and epidemiological quantitative data, and in-depth qualitative interviews, creates a comprehensive approach, which is particularly important when trying to understand issues of influenza vaccine compliance and hesitancy. Finally, the study cohort includes several unique sub-studies.

### Limitations

Our study has at least four limitations. First, our ability to generalize vaccine effectiveness findings from the study years to the potential preventive value of an influenza vaccine program may be limited. The effectiveness of the vaccine depends in part on the types of viruses circulating and the antigenic and genetic match between vaccine components and circulating strains in a given year. The precision of the estimates will also depend on the number of influenza cases.

Second, we will be cautious in interpreting self-reported information, given potential biases in recall and self-presentation. While some of the information collected through active surveillance can be verified with administrative data, other information, such as symptoms for non-medically attended illnesses, cannot be verified by another data source.

Third, although the random stratified sampling design intentionally includes HCP with a mixture of characteristics and work responsibilities, there are relatively few HCP in some of the strata. For example, few male medical assistants and support personnel are employed at the two hospitals, and therefore it is unlikely that we will be able to enroll large numbers from this category. This may limit our ability to examine the association between the frequency of influenza illness and specific combinations of age, sex, and occupation.

Fourth, during the third wave of recruitment we accept volunteers, regardless of age, profession, or vaccine status, which can present a sampling bias. However, characteristics of these volunteer participants will be compared with those of participants recruited during the first two waves in order to evaluate potential differences between the groups.

## Additional file

Additional file 1: Description of additional terms and methods used in the Study of Healthcare Personnel with Influenza and other Respiratory Viruses in Israel. (DOCX 77 kb)

## Abbreviations

ARFI: Acute respiratory illness or febrile illness
CHS: Clalit Health Services
CVL: Clinical Virology Laboratory
EMR: Electronic medical record
GMT: Geometric mean titer
GMR: Geometric mean ratios
HCP: Healthcare personnel
HI: Hemagglutination inhibition
hMPV: Human metapneumovirus
ICD-9: The international classification of diseases, ninth revision
ICPC: International Classification of Primary Care Coding
IVE: Influenza vaccine effectiveness
KAP: Knowledge, attitudes, and practices
mqRT-PCR: Multiplex quantitative real-time reverse transcription polymerase chain reaction
PBMC: Peripheral blood mononuclear cells
RBC: Red blood cells
REDCap: Research Electronic Data Capture
RSV: Respiratory syncytial virus
SHIRI: Study of Healthcare Personnel with Influenza and other Respiratory Viruses in Israel
SMS: Short message service
US CDC: United States Centers for Disease Control and Prevention
