# Supplementary material for: Study of Healthcare Personnel with Influenza and other Respiratory Viruses in Israel (SHIRI): study protocol
Source: BMC Infect Dis. 2018 Nov 6;18:550. doi: 10.1186/s12879-018-3444-7 (PMC6220521; doi:10.1186/s12879-018-3444-7)
Supplement: Supplementary file 1 — Description of additional terms and methods used in the Study of Healthcare Personnel with Influenza and other Respiratory Viruses in Israel. (DOCX 77 kb) [file 12879_2018_3444_MOESM1_ESM.docx]

**Study of Healthcare Personnel with Influenza and other Respiratory Viruses in Israel (SHIRI): Study Design and Methods**

**Supplementary file**

**Annex 1. Defining chronic medical conditions**

We identified a broad list of chronic medical conditions, including conditions that have been previously identified as risk factors for severe outcomes from influenza infections by the US Advisory Committee on Immunization Practices (ACIP) (1). In order to fully characterize the health profiles of participants, we also identified chronic diseases not included in ACIP risk groups, such as cancer, chronic gastrointestinal disease, and immunocompromised conditions.

We will use a combination of methods to identify these chronic conditions. We will include data linked to participants’ outpatient and inpatient medical encounters during the year prior to the relevant study year. To determine relevant codes for chronic medical condition, we used ICD-9 codes described in previously published influenza studies (Naleway et al, pregnancy methods, in press)(2) along with ICD-9 codes that have been used previously to describe chronic medical conditions among CHS members. We will also use relevant International Classification of Primary Care (ICPC) codes (3), which are used in CHS for outpatient visits exclusively, and we will review free text diagnoses recorded by physicians during participants’ medical visits. ICD-9 codes, ICPC codes, and other data sources that will be used to determine chronic medical conditions are listed in Table 1. All chronic medical conditions will be extracted as of September 1 of the relevant study year. A participant will be considered to have a specific medical condition if the specific chronic medical condition was reported during an inpatient or outpatient visits at least one time in the previous year.

We also will identify chronic medical conditions recorded through other relevant databases and algorithms which include data that are not specific to previous year medical visits. For example, CHS has a number of chronic disease registries, which compile patient data for a number of chronic diseases (See Table 1 for relevant diseases identified through the chronic disease registry). We will identify patients with diabetes through the use of a validated algorithm developed by the Clalit Research Institute that incorporates multiple clinical criteria (4). For chronic kidney disease, we will use an internal algorithm that uses clinical and demographic data to identify patients who have chronic kidney disease according to the classification scheme outlined by the US National Kidney Foundation (5). Finally, we will extract cancer data from the Israel Ministry of Health National Cancer Registry that has been merged into the CHS EMRs.

In order to identify immunocompromised patients, we used an algorithm that was adapted from a recently published algorithm to identify immunocompromised patients as part of a study of Herpes Zoster infections in Kaiser Permanente members (6).We simplified the algorithm so that patients were simply dichotomized into “immunocompromised” or “non-immunocompromised.” The algorithm is explained in Annex 2.

Table 1. Data sources used for the identification of chronic medical conditions for the SHIRI study, Israel.

| Chronic Disease | Relevant ICD-9 codes | Relevant ICPC codes | Other data sources |
| --- | --- | --- | --- |
| Hematologic Disorders | ICD-9 281-289 | B71, B81, B83, B78, B82 | Chronic disease registry for pernicious anemia; hemophilia; thalassemia; sickle cell anemia; other hematologic disorders |
| Diabetes |  |  | CRI algorithm (4) |
| Chronic cardiovascular disease | 250.7, 391, 394, 395, 396, 397, 398, 410-414, 424.0, 424.1, 427.3, 427.31, 427.32, 428, 430-438, 440, 442, 444.2, 445.0, 746, I25.5, I42, I50, I60.0-9, G45.9 | K71, K75, K76, K78, K92, K90, K83 | Chronic disease registries for status/post stroke; cardiac arrhythmia; ischemic heart disease; valvular heart disease (except mitral valve prolapse); congestive heart failure |
| Cancer |  |  | Israel Ministry of Health Cancer Registry |
| Chronic Kidney Disease |  |  | CRI algorithm for chronic kidney disease, based on laboratory and demographic data |
| Liver Disease | 571, 572 | D97 | Chronic disease registry for cirrhosis, other liver disease |
| Gastrointestinal Disease | 530, 531, 532, 533, 544, 555, 556, 579.0 | D86, D94, | Chronic disease registry for peptic ulcer disease; celiac disease; ulcerative colitis; crohn’s disease; Refluex Esophagitis/Gastritis/Duodenitis |
| Chronic Pulmonary Disease | 11, 12, 13, 135, 277.0, 491.2, 492, 493,494, 495, 496, 500, 501, 502, 503, 504, 505, 506, 508, 514, 515, 516, 517, 518, 519, 748 | R70, R95 | Chronic Disease Registries for COPD and asthma |
| Metabolic, Nutritional and Endocrinologic (excluding Diabetes) | 240, 241, 242, 243, 244, 245, 246, 252.0, 253, 254, 255, 260, 261, 262, 263, 264, 265, 266, 267, 268, 269, 270, 271, 273, 274, 275, 277.1-9 | T81, T85, T86, T91 | Chronic Disease Registry for hyperthyroidism, hypothyroidism, hypo/hyperparathyroidism, acromegaly, Cushing’s Disease, |
| Neuromuscular | 013, 046, 137.1, 290, 294, 331, 310, 318, 330, 331, 332, 333, 334, 335, 336, 337, 340, 345, 348, 349, 358, 359, 392, 430, 431, 432, 436, 437, 438, 728, 756, 780.3, 780.72, 907.2, 996.2, 996.75 | N88 | Chronic Disease Registry for Parkinson’s Disease, Cerebral Palsy, Multiple Sclerosis, Myesthenia Gravis, Muscular Dystrophy |
| Immunocompromised |  |  | See Annex 2 |

**Annex 2. Determining Immunocompromised Status (Adapted from algorithm published in Baxter et al.(6))**

**Measures of Immunocompromised Status**

Immunocompromised (IC) status is measured on September 1 of the relevant study year.

**IC status at time of risk**

We used eight variables to measure IC status, based primarily on conditions and medications during the past six or 12 months.

For each of the following variables, the person was assigned “1” to designate an immunocompromised state if the condition or medication was present, or “0” if it was not present:

1. Corticosteroids
2. Immunocompromising medications other than corticosteroids
3. Cancer radiotherapy
4. Blood cancer
5. Metastatic cancer
6. Bone marrow transplant (BMT) or hematopoietic stem cell transplant (HSCT)
7. Human Immunodeficiency Virus (HIV)
8. Rare immune deficiency conditions

The eight variables are not mutually exclusive (i.e., persons could be designated immunocompromised in more than one category). Individuals were considered immunocompromised if any one of the eight conditions were present.

**Data sources**

Data sources included electronic medical records from CHS, including all records from CHS clinics, hospitals, and pharmacies. We included medications purchased from a CHS pharmacy or hospital, and identified conditions from outpatient, emergency department, and inpatient diagnoses. We used both primary and secondary diagnoses.

We also used the Israel Ministry of Health Cancer Registry as a source for identifying persons with cancer and individuals who initially presented with metastatic cancer.

**Eight variables used to measure IC status**

Five of the eight IC variables indicate the presence of conditions or medications during the 12-month period prior to September 1 of the relevant study year. For corticosteroids, other immunocompromising medications, and cancer radiotherapy, only the previous six-month period was considered.

**1. IC** – **Corticosteroids**
We counted oral or intravenous forms of the following corticosteroids: prednisone, prednisolone, hydrocortisone, methylprednisolone, betamethasone, cortisone, triamcinolone, and dexamethasone. We considered patients immunocompromised if they used any amount of these medications during the previous six months. We based this definition, like the other immunocompromised categories, on an algorithm for defining immunocompromised patient cohorts developed by Kaiser and Merck in an article published in 2017 in the American Journal of Epidemiology (6). While definitions of the amount of steroid use constituting immunocompromised status varies, short-term low-dose corticosteroid use has been associated with a number of moderate and severe adverse events(7).

**2. IC – Immunocompromising medications other than corticosteroids**

We considered non-corticosteroid immunocompromising medications during the six months prior to September 1 of the relevant study year. The list of immunocompromising medications included therapeutic categories known to inhibit immune response, including anti-neoplastic (chemotherapy), disease modifying anti-rheumatic (DMARD), immunomodulator agents, and other immunosuppressant medications. Use of any of these medications at least once during the previous six months conferred immunocompromised status. These medications are described in Table 5 are classified according to immunocompromising status (0 = No IC, 1 = IC). As above.

**3. IC – Cancer radiotherapy**

We considered radiation treatment in the six months prior to September 1 of the relevant study year to confer immunocompromised status. We included diagnosis and procedure codes as well as data from radiation oncology visits.

We identified radiation treatment administered by CHS using ICD-9 diagnosis codes (Table 1) and relevant CPT-4 codes and other procedural codes (V580, V661, V671).

**Table 1. Diagnosis and procedure codes for cancer radiotherapy**

| **ICD-9** | **Description** |
| --- | --- |
| V58.0 | Encounter for radiotherapy |
| V66.1 | Convalescence following radiotherapy |
| V671 | Follow-up examination following radiotherapy |

**4. IC** – **Blood Cancer**

Persons were considered immunocompromised if they were diagnosed as having blood cancer (ICD-9 codes 200-208 or similar diagnoses in the Israel Ministry of Health cancer registry) during the previous year (Table 2).

**Table 2. Categories of blood cancers (ICD-9 codes 200-208)**

| **ICD-9** | **Description** |
| --- | --- |
| 200 | Lymphosarcoma and reticulosarcoma and other specified malignant tumors of lymphatic tissue |
| 201 | Hodgkin's disease |
| 202 | Other malignant neoplasms of lymphoid and histiocytic tissue |
| 203 | Multiple myeloma and immunoproliferative neoplasms |
| 204 | Lymphoid leukemia |
| 205 | Myeloid leukemia |
| 206 | Monocytic leukemia |
| 207 | Other specified leukemia |
| 208 | Leukemia of unspecified cell type |

**5. IC – Metastatic cancer**

We used staging information from the Israel Ministry of Health cancer registry to identify persons with metastatic disease at initial diagnosis. In the registry, persons are classified as having metastatic cancer if either Surveillance, Epidemiology, and End Results (SEER) or Tumor, Nodes, Metastasis (TNM) staging indicates an advanced stage or the presence of distant metastases.

We used metastasis diagnosis (ICD-9 codes 196-199) to identify persons who develop metastasis at a later point. (The Israel Ministry of Health cancer registry includes all newly diagnosed cases of cancer but does not capture information on recurrences or progression to metastatic cancer).

Persons identified as having metastatic cancer from the Israel Ministry of Health cancer registry were considered to have metastatic cancer if they were listed in the registry as having metastatic cancer. Persons identified as having metastatic cancer through an ICD-9 diagnosis alone were only considered to have metastatic cancer if the ICD-9 code was recorded in the previous year. This distinction reflects the higher accuracy of the Israel Ministry of Health registry compared to diagnosis codes for identifying metastatic cancer.

**6. IC – Bone marrow or hematopoietic stem cell transplant (BMT/HSCT)**

Persons were considered immunocompromised if they received a diagnosis during the past year indicating that they have had a BMT or HSCT at any time. We identified a mix of transplant recipients who received their transplants in the recent and distant past.

We used ICD-9 procedure codes and equivalent CHS procedure codes to find allogeneic BMT and HSCT procedures both allogeneic and autologous. (Table 3)

**Table 3. Diagnosis and procedure codes for BMT and HSCT**

| **ICD-9** | **Description** |
| --- | --- |
| Diagnosis codes: | |
| V42.81 | Bone marrow replaced by transplant |
| V42.82 | Peripheral stem cells replaced by transplant |
| 996.85 | Complications of transplanted bone marrow |
| Procedure codes: | |
| 41.02 | Allogeneic bone marrow transplant with purging |
| 41.03 | Allogeneic bone marrow transplant without purging |
| 41.05 | Allogeneic hematopoietic stem cell transplant without purging |
| 41.08 | Allogeneic hematopoietic stem cell transplant with purging |

**7. IC** – **HIV**

Persons were considered immunocompromised they had record of HIV infection, regardless of CD4 count or medications.

**8. IC – Rare immune deficiency conditions**

Persons were considered immunocompromised if they had selected diagnoses from a number of categories of immune disorders in the year prior to September 1 of the relevant study year. The descriptions of the diseases and the relevant ICD-9 codes are listed in Table 4.

**Table 4. Diagnosis codes for rare immune deficiency conditions and the IC level assigned to each diagnosis to measure IC status at vaccination**

| **ICD-9** | |  |
| --- | --- | --- |
| **Code** | **Description** |  |
| 279.0x | Deficiency of humoral immunity |  |
| 279.10 | Immunodeficiency with predominant T-cell defect, unspecified |  |
| 279.11 | Digeorge's syndrome |  |
| 279.12 | Wiskott-aldrich syndrome |  |
| 279.13 | Nezelof's syndrome |  |
| 279.19 | Other deficiency of cell-mediated immunity |  |
| 279.2 | Combined immunity deficiency |  |
| 279.3 | Unspecified immunity deficiency |  |
| 279.41 | Autoimmune lymphoproliferative syndrome |  |
| 284.09 | Other constitutional aplastic anemia |  |
| 284.89 | Other specified aplastic anemias |  |
| 284.9 | Aplastic anemia, unspecified |  |
| 288.01 | Congenital neutropenia |  |
| 288.1 | Functional disorders of polymorphonuclear neutrophils |  |
| 288.2 | Genetic anomalies of leukocytes |  |

**Table 5. List of immunocompromising medications other than corticosteroids with IC level assignments**

| **Generic Drug Name** | **IC Classification Assignment**  (0= no IC, 1= IC) |
| --- | --- |
| Abatacept | 1 |
| Abiraterone | 0 |
| Adalimumab | 1 |
| Ado-Trastuzumab Emtansine | 1 |
| Afatinib | 0 |
| Aldesleukin | 1 |
| Alectinib | 0 |
| Alemtuzumab | 1 |
| Alitretinoin | 1 |
| Altretamine | 1 |
| Aminocamptothecin | 0 |
| Aminoglutethimide | 1 |
| Amonafide | 1 |
| Anakinra | 1 |
| Anastrozole | 0 |
| Antithymocyte Globulin | 1 |
| Apremilast | 1 |
| Arsenic Trioxide | 1 |
| Asparaginase | 1 |
| Auranofin | 1 |
| Axitinib | 0 |
| Azacitidine | 1 |
| Azathioprine | 1 |
| Basiliximab | 1 |
| BCG Vaccine | 1 |
| Belatacept | 1 |
| Belinostat | 1 |
| Bendamustine | 1 |
| Bevacizumab | 1 |
| Bexarotene | 1 |
| Bicalutamide | 0 |
| Bleomycin | 1 |
| Blinatumomab | 1 |
| Bortezomib | 1 |
| Bosutinib | 1 |
| Brentuximab Vedotin | 1 |
| Busulfan | 1 |
| Cabazitaxel | 1 |
| Cabozantinib | 1 |
| Capecitabine | 1 |
| Carboplatin | 1 |
| Carfilzomib | 1 |
| Carmustine | 1 |
| Ceritinib | 1 |
| Certolizumab Pegol | 1 |
| Cetuximab | 1 |
| Chlorambucil | 1 |
| Cisplatin | 1 |
| Cladribine | 1 |
| Clofarabine | 1 |
| Cobimetinib | 1 |
| Crizotinib | 1 |
| Cyclophosphamide | 1 |
| Cyclosporine | 1 |
| Cytarabine | 1 |
| Dabrafenib | 1 |
| Dacarbazine | 1 |
| Daclizumab | 1 |
| Dactinomycin | 1 |
| Daratumumab | 1 |
| Dasatinib | 1 |
| Daunorubicin | 1 |
| Decitabine | 1 |
| Degarelix | 0 |
| Denileukin Diftitox | 1 |
| Dexrazoxane | 0 |
| Dimethyl Fumarate | 1 |
| Dinutuximab | 1 |
| Docetaxel | 1 |
| Doxorubicin | 1 |
| Eculizumab | 1 |
| Efalizumab | 1 |
| Elotuzumab/Elotozumab | 0 |
| Enzalutamide | 0 |
| Epirubicin | 1 |
| Eribulin | 1 |
| Erlotinib | 1 |
| Estramustine | 1 |
| Etanercept | 1 |
| Etoposide | 1 |
| Everolimus | 1 |
| Exemestane | 0 |
| Filgrastim | 1 |
| Floxuridine | 1 |
| Fludarabine | 1 |
| Fluorouracil | 1 |
| Flutamide | 0 |
| Fulvestrant | 0 |
| Gefitinib | 1 |
| Gemcitabine | 1 |
| Gemtuzumab | 1 |
| Gold Sodium Thiomalate | 0 |
| Golimumab | 1 |
| Goserelin | 0 |
| Histrelin | 0 |
| Hydroxychloroquine Sulfate | 0 |
| Hydroxyurea | 1 |
| Ibrutinib | 1 |
| Idarubicin | 1 |
| Idelalisib | 1 |
| Ifosfamide | 1 |
| Imatinib Mesylate | 1 |
| Infliximab | 1 |
| Inosine Pranobex | 1 |
| Interferon (All Types) | 1 |
| Ipilimumab | 0 |
| Irinotecan | 1 |
| Isotretinoin | 1 |
| Ixabepilone | 1 |
| Ixazomib | 1 |
| Lanreotide | 0 |
| Lapatinib | 0 |
| Leflunomide | 1 |
| Lenalidomide | 1 |
| Lenvatinib | 0 |
| Letrozole | 0 |
| Leuprolide | 0 |
| Lomustine | 1 |
| Mechlorethamine | 1 |
| Megestrol Acetate | 1 |
| Melphalan | 1 |
| Mercaptopurine | 1 |
| Methotrexate (oral) | 1 |
| Methotrexate (injection) | 1 |
| Methoxsalen | 1 |
| Mitomycin | 1 |
| Mitotane | 1 |
| Mitoxantrone | 1 |
| Muromonab-CD3 | 1 |
| Mycophenolate | 1 |
| Necitumumab | 0 |
| Nelarabine | 1 |
| Nilotinib | 1 |
| Nilutamide | 0 |
| Nivolumab | 0 |
| Obinutuzumab | 1 |
| Octreotide | 0 |
| Ofatumumab | 1 |
| Olaparib | 1 |
| Omacetaxine | 1 |
| Oprelvekin | 0 |
| Osimertinib | 1 |
| Oxaliplatin | 1 |
| Paclitaxel | 1 |
| Palbociclib | 1 |
| Panitumumab | 1 |
| Panobinostat | 1 |
| Pazopanib | 1 |
| Pegaspargase | 1 |
| Pegfilgrastim | 1 |
| Peginterferon | 1 |
| Pembrolizumab | 0 |
| Pemetrexed | 1 |
| Penicillamine | 1 |
| Pentostatin | 1 |
| Pertuzumab | 0 |
| Pomalidomide | 1 |
| Ponatinib | 1 |
| Porfimer | 0 |
| Pralatrexate | 1 |
| Procarbazine | 1 |
| Ramucirumab | 0 |
| Regorafenib | 0 |
| Rituximab | 1 |
| Romidepsin | 1 |
| Ruxolitinib | 1 |
| Sargramostim | 1 |
| Siltuximab | 0 |
| Sipuleucel-T | 0 |
| Sirolimus | 1 |
| Sonidegib | 0 |
| Sorafenib | 1 |
| Streptozocin | 1 |
| Sulfasalazine | 0 |
| Sunitinib | 1 |
| Tacrolimus | 1 |
| Talimogene Laherparepvec | 0 |
| Tamoxifen | 0 |
| Temozolomide | 1 |
| Temsirolimus | 1 |
| Teniposide | 1 |
| Testolactone | 0 |
| Thalidomide | 1 |
| Thioguanine | 1 |
| Thiotepa | 1 |
| Tocilizumab | 1 |
| Tofacitinib | 1 |
| Topotecan | 1 |
| Toremifene | 0 |
| Trabectedin | 1 |
| Trametinib | 0 |
| Trastuzumab | 1 |
| Tretinoin | 1 |
| Trifluridine a+A164nd Tipiracil | 1 |
| Trimetrexate | 1 |
| Triptorelin | 0 |
| Ustekinumab | 1 |
| Valrubicin | 1 |
| Vandetanib | 1 |
| Vemurafenib | 0 |
| Vinblastine | 1 |
| Vincristine | 1 |
| Vinorelbine | 1 |
| Vismodegib | 0 |
| Vorinostat | 1 |
| Ziv-Aflibercept | 0 |

*This list of medications is identical to the list published in Baxter et al.

References

1. Grohskopf LA, Sokolow LZ, Broder KR, Walter EB, Bresee JS, Fry AM, et al. Prevention and Control of Seasonal Influenza with Vaccines: Recommendations of the Advisory Committee on Immunization Practices — United States, 2017–18 Influenza Season. MMWR Recomm Reports. 2017 Aug;66(2):1–20.

2. Jackson ML, Phillips CH, Benoit J, Jackson LA, Gaglani M, Murthy K, et al. Burden of medically attended influenza infection and cases averted by vaccination – United States, 2013/14 through 2015/16 influenza seasons. Vaccine. 2017 Dec;

3. Wonca. ICPC-2 – EnglishInternational Classification ofPrimary Care – 2nd EditionWonca InternationalClassification Committee(WICC).

4. Karpati T, Cohen-Stavi CJ, Leibowitz M, Hoshen M, Feldman BS, Balicer RD. Towards a subsiding diabetes epidemic: trends from a large population-based study in Israel. Popul Health Metr. 2014;12(1):32.

5. Levey AS, Coresh J, Balk E, Kausz AT, Levin A, Steffes MW, et al. National Kidney Foundation practice guidelines for chronic kidney disease: evaluation, classification, and stratification. Ann Intern Med. 2003 Jul;139(2):137–47.

6. Baxter R, Bartlett J, Fireman B, Marks M, Hansen J, Lewis E, et al. Long-Term Effectiveness of the Live Zoster Vaccine in Preventing Shingles: A Cohort Study. Am J Epidemiol. 2017 Jun;1–9.

7. Waljee AK, Rogers MAM, Lin P, Singal AG, Stein JD, Marks RM, et al. Short term use of oral corticosteroids and related harms among adults in the United States: population based cohort study. BMJ. 2017;357:j1415.

**Annex 3 – ICD-9 codes and ICPC Codes Used to Define Acute Respiratory and Febrile Illness**

| **Description** | **ICD-9 Code** | **ICPC Code** |
| --- | --- | --- |
| Respiratory syncytial virus (RSV) | 79.6 | - |
| Viral infection, unspecified | 79.99 | - |
| Acute nasopharyngitis (common cold) | 460 | - |
| Acute sinusitis | 461 | R75 |
| Acute pharyngitis | 462 | - |
| Acute tonsillitis | 463 | R22 |
| Acute laryngitis and tracheitis | 464 | R77 |
| Acute upper respiratory infections of multiple or unspecified sites | 465 | - |
| Acute bronchitis and bronchiolitis | 466 | R78 |
| Viral pneumonia | 480 | - |
| Pneumococcal pneumonia | 481 | - |
| Other bacterial pneumonia | 482 | - |
| Pneumonia due to other specified organism | 483 | - |
| Pneumonia in infectious diseases classified elsewhere | 484 | - |
| Bronchopneumonia, organism unspecified | 485 | - |
| Pneumonia, organism unspecified | 486 | R81 |
| Influenza | 487 | R80 |
| Influenza due to identified novel influenza A virus | 488 | - |
| Bronchitis, not specified as acute or chronic | 490 | - |
| Pulmonary collapse | 518 | - |
| Pulmonary edema | 518.4 | - |
| Acute respiratory failure | 518.81 | - |
| Unspecified disease of respiratory system | 519 | - |
| Fever, unspecified + Cough | 780.60 + 786.2 | - |
| Chills (without fever) | 780.64 | - |
| Other malaise and fatigue | 780.79 | - |
| Respiratory abnormality, unspecified | 786 | - |
| Shortness of breath | 786.05 | - |
| Tachypnea | 786.06 | - |
| Wheezing | 786.07 | - |
| Respiratory abnormality, other | 786.09 | - |
| Stridor | 786.1 | - |
| Abnormal sputum | 786.4 | - |
| Painful respiration | 786.52 | - |
| Abnormal chest sounds | 786.7 | - |
| Hypoxemia | 799.02 | - |

**Annex 4 – Hemagglutination Inhibition Assay**

The Hemagglutination Inhibition (HAI) Assay is used to detect the presence of neutralizing influenza antibodies in serum. Specifically, HAI assay measures the ability of anti-hemagglutinin (HA) antibodies to inhibit the binding of HA viral protein with sialic acid (SA) on the surface of the red blood cells (RBCs) (erythrocyte) which causes the cells to agglutinate *in vitro*. Inhibition of this agglutination by antibodies which neutralize the binding of HA with erythrocytes forms the basis of the HAI assay.

HAI to IIV components and to influenza circulating strains during the study period will be performed by Battelle Laboratory (Aberdeen, MD) using egg-grown wild type viruses supplied by US CDC’s International Reagent Resource. Ether treated type B influenza antigens will be used given evidence of increased ability to detect HAI following ether treatment.

Preparation of serum samples for us in the HAI assay includes the following three steps. First, serum samples will be treated with receptor destroying enzyme to remove non-specific inhibitors (various sialic acid-containing glycans) that may mimic the binding of influenza-specific anti-HA antibodies. Second, serum samples will be tested for non-specific agglutination, which may cause false negative HAI results. When non-specific agglutinins are observed (when diluted serum and a solution of RBCs are combined and hemagglutination occurs), serum must be adsorbed with RBC before HAI testing. Serum samples with RBC agglutination and point titers of 80 or higher will be adsorbed and tested again for non-specific agglutination prior to testing in the HAI assay to confirm the removal of nonspecific agglutinins. If the end point titer is still ≥20 after adsorption, then they must be adsorbed again.

The choice of RBCs to use in the HAI assay is dependent upon the virus being tested. HAI is often performed with turkey RBCs since they are large, nucleated, and sediment and quickly allow determination of an HAI endpoint. HAI against A(H1N1)pdm09 and B influenza antigens will be conducted with turkey RBCs. With new antigenic clusters of A(H3N2) since 2014, the neuraminidase (NA) of these viruses have acquired the ability to bind to RBCs. For these viruses, modified HAI assays will be conducted using Guinea Pig red blood cells in the presence of the antiviral oseltamivir carboxylate, which inhibits influenza NA.

Serum will be diluted 2-fold starting from 1:10. The serum HAI titer is the reciprocal of the dilution of virus in the last well with complete hemagglutination inhibition. The reported HAI titer of a sera sample is the geometric mean titer (GMT) from duplicate assays run on separate plates. HAI <10 will be considered 5 for GMT calculation. If the results are not within one 2-fold serial dilution of each other, then the assay is repeated. If a test sample has a HAI titer of ≥1280, the test sample must be repeated at a higher starting dilution until the end-point titer is obtained.

References

1. Network WHOGIS. Serological diagnosis of influenza by haemagglutination inhibition testing. In: Manual for the laboratory diagnosis and virological surveillance of influenza. Geneva, Switzerland: WHO, 2011.

2. Gaglani M, Spencer S, Ball S, et al. Antibody response to influenza A(H1N1)pdm09 among healthcare personnel receiving trivalent inactivated vaccine: effect of prior monovalent inactivated vaccine. The Journal of infectious diseases **2014**; 209(11): 1705-14.

3. Thompson MG, Naleway A, Fry AM, et al. Effects of Repeated Annual Inactivated Influenza Vaccination among Healthcare Personnel on Serum Hemagglutinin Inhibition Antibody Response to A/Perth/16/2009 (H3N2)-like virus during 2010-11. Vaccine **2016**; 34(7): 981-8.

**Annex 5 – Interview Guide for Qualitative Interviews with Participants Regarding Attitudes toward Morbidity from Influenza and Respiratory Illness**

The following list of questions and probes (or follow-up sub-questions) will serve as a guide for the topics to discuss in in-depth qualitative interviews to be conducted with a sub-sample of consented HCPs. Because these will be qualitative interviews, the exact phrasing and the order of the questions may be adapted for each interview, depending on the flow of the discussion.

Interviewers should be aware of the dates of the HCP’s illness, the test results of the respiratory sample(s) in all years of the study, and the vaccination status of the HCP in all years of the study.

1. Please describe your experience with your recent illness.

Probes:

- What symptoms did you experience?
- How long did you feel ill?
- How sick did you feel? How severe was your illness?
- How did your illness impact your daily activities and responsibilities?

2. How did the illness impact your work responsibilities?

Probes:

- When did you first suspect you were ill and was this at work or outside of work?
- Did you miss work or reschedule work hours due to the illness?
- Did you come to work at all when you were sick? If so, why?
- Do you feel pressure not to miss work, even if that means coming to work while sick? If so where does the pressure come from?
- What factors impact your decision to go to work or miss work when you are sick?
- Does your organization encourage you to go home if you are at work and feel sick with a respiratory illness? How?
- Does your organization discourage you from missing work when you feel sick with a respiratory illness? How?
- On your sickest day, from 0 to 9 (0 being not able to 9 being extremely able), how would you rate your ability to execute your work responsibilities?

3. Please describe how the respiratory illness impacted your life outside of work and usual activities (at work and at home).

Probes:

- What part of your life was most impacted by your illness?
- What usual activities of yours were most impacted by your illness?
- Did any disruption to your home or personal life associated with the illness in turn impact your work responsibilities?
- On your sickest day, from 0 to 9 (0 being not able to 9 being extremely able), how would you rate your ability to execute your responsibilities at home?

4. Will your recent respiratory illness this year affect your decision to get or not get the influenza vaccine in general?

Probes:

- Does the fact that your recent respiratory sample from the study was positive for flu [if infected]/negative for flu [if not infected] this season change your opinion on influenza and influenza vaccination? How about the results from your respiratory specimen from last year (if applicable)?
- Did you get vaccinated before this influenza season? Will you get vaccinated next year?
- Is your decision to get vaccinated against influenza impacted by your recent illness and/or influenza infection this year or last year?
- What are the reasons why you chose to get vaccinated (or not vaccinated) with the flu vaccine this season?
- How did your recent illness last year (if you were ill) compare with your illness experience this year?

**Annex 6 - TextIt**

**Technology Overview**

A platform was designed and developed to conduct routine influenza-like-illness surveillance via SMS messaging to support the longitudinal data collection needs. Built on the TextIt platform, logic messaging flows were constructed that track illness events, provide personalized reminders to participants, and alert study staff of critical events and participants’ needs through automated emails. Based on participant responses, the system saves key variables to a participants’ contact profile on the TextIt platform, such as date of reported illness.  The system then generates customized follow-up messages based on these unique variables in order to track each participant’s duration of illness. Routine data exports are conducted by in-country staff and imported into macro-enabled Excel workbook tools for capture of key variables. For implementation of SMS surveillance, Twilio long virtual numbers were purchased to support message sending and receiving from the TextIt platform. Twilio is a cloud communications platform as a service (PaaS) company that provides infrastructure APIs for businesses to build scalable, reliable voice and text messaging apps.

A corresponding database was developed using the REDCap platform in order to collect enrollment and illness data on participants via online interviews and surveys. In-country data management teams export survey links from REDCap and upload to TextIt in order to send unique survey links to enrolled participants via SMS.

**What data is being captured/stored by TexIt**

Routine influenza-like-illness surveillance data are captured via SMS interactions with surveys programmed using the TextIt platform. The data captured and stored for each participant in the study focus on cataloguing suspected influenza illness events, information on symptoms experienced, duration of illness, and key pieces of data relating to Objective 1, including: missed work due to influenza illness, and hours of direct patient care provided by HCP with symptomatic influenza infections. Data are stored in multiple formats: numeric, text, and date & time. All data are captured from the TextIt messaging logic flows and stored within each study participant’s profile as a contact variable. Since these data overwrites with each round of bi-weekly surveillance, study staff must perform bi-weekly exports of TextIt data and import the data into an Excel macro-enabled workbook in order to track participant responses and illness events over time.

**Database**

As the TextIt platform is designed to be a longitudinal data repository, sites have macro-enabled Excel workbook tool where they store TextIt data over time. Database managers are required to export TextIt flow and contact data *prior to the start of each active surveillance messaging round* in order to capture the most recent messaging events data and catalog it over time. Because we use the same messaging flows in cycles, each time someone responds, their prior response will be overwritten. Thus, we need to export the data right before the next messaging round in order to capture data for each individual messaging round scheduled in the system.

**How are the data used?**

The SMS data captured during routine influenza-like-illness surveillance are used in real-time to direct data collection activities on an hourly basis for the project. Because we are tracking illness events, timely surveying unique to each participant’s condition is critical to ensure up-to-date information and to reduce recall biases amongst participants. There are a number of surveys conducted depending on illness status of a participant, each of which is governed by a detailed protocol—for example, if a participant is illness free they are to receive routine messaging twice per week asking whether they have experienced influenza-like symptoms within the past 7 days. If a participant is currently ill, data are collected (i) to ascertain the duration of their illness on set time intervals, (ii) to assess whether biological specimens have been collected and delivered to study staff, and (iii) to evaluate the impact of an illness upon resolution pertaining to the number of work hours missed, etc. The TextIt SMS flows are critical to capturing appropriate data at key time points throughout project implementation.

**SMS Message Flows:**

A “flow” is a series of steps create the ‘interaction’ that our study participants experience.

- In general, the flows include:
  - Routine Surveillance Flows – ascertain suspected influenza events; capture details on illness event
  - Acute Illness Surveys – capture granular information on the types of symptoms experienced shortly after notice of an illness event through Routine surveillance
  - Follow-up Illness messaging: To ascertain how long the illness is experienced
  - Reminders: to follow-up on missed responses; to remind participants to take nasal swab samples and transport to the lab
- Flows are implemented in the native language (Hebrew).
- Flows have built in logic to ensure participants receive the appropriate messaging at the appropriate time.

**Channels and Message Phasing Overview**

TextIt requires a “channel,” which allows for sending and receiving messages or phone calls through the TextIt account. As many channels can be connected to the TextIt account as needed. The channel used for this project was the SMS aggregator Twilio. Aggregators are services that process messages across multiple carriers with exceptional speed and efficiency. They provide **virtual mobile numbers**and **short codes** that are easily connected to TextIt accounts through an API integration. Messages are sent through local mobile network operators (MNO). As a result, the system can be challenged by MNO throttling due to the large volumes of messages that are sent at one time (i.e., a routine active surveillance messaging round on Sundays of each week). As a result, implementation followed a phased approach, where the participants were partitioned into groups in two ways. First, multiple channels, or phone numbers, were connected to each account. The data management staff then assigned participants to a specific channel within each account upon importing into the system. Second, participants were grouped into different ‘time slots’ throughout the day to receive their messages. Typically, there are three time slots a day that participants will be assigned to (through a Date&Time contact variable). Thus, for example when it comes time for an active messaging round, all participants will still receive their messages on the required day, but the messages will be sent in blocks (re: 10 AM, 1 PM, 3 PM) so as to limit the volume of messages even further from each of the channels.

**Annex 7 – Statistical Considerations**

**Sample Size for VE and Incidence Objectives**

We estimated that with 2,430 participants (contributing 5 months at-risk of influenza infection per season or 1,012 participant years), 40% vaccine coverage, and 10% influenza illness attack rate, we would be powered to estimate a true VE of 50% with confidence intervals that do not overlap with zero. Assuming within season attrition of ~9%, a cohort of approximately 2,800 HCP would ensure an analytic sample of >2,300.

Kuster et al.’s meta-analysis of HCP studies [[1](file:///C:\Users\avitalhi\AppData\Local\Microsoft\Windows\Temporary%20Internet%20Files\Content.Outlook\O9HTX1CP\HCP%20Israel_Methods%20Paper_Statistical%20Considerations_vs1_072417.docx#_ENREF_1)] estimated the incidence of symptomatic influenza infection in a typical influenza season at 7.5 per 100 unvaccinated HCP and 4.8 per 100 vaccinated HCP. This is lower than the 10% attack rate assumed in our preliminary power analyses. However, our study design involves a broader case definition and intensive surveillance, which should optimize influenza detection. Nonetheless, we may be underpowered to estimate VE if the season duration, attack rate, or vaccination coverage is lower than expected. Combining results with the related US-CDC-funded HCP cohort study in Peru and/or a multi-season model may be required to examine whether VE varied by age, prior vaccination exposure, occupation, or other potential effect modifiers.

Additional scenarios relative to sample size planning were considered and are summarized in Table 1. These scenarios included the following assumptions:

- Each participant contributes approximately five months at-risk for influenza infection; thus, 2,430 participants would contribute 1,012 total participant-years (p-yrs);
- Background incidence rates of 5% (5/100 p-yrs) and 10% (10/100 p-yrs) were considered;
- Unadjusted VE estimates of 40% and 50% were examined.

Under these assumptions, a cohort of 2,430 would be able to detect differences in influenza incidence between vaccinated and unvaccinated participants except in situations where the background incidence was low (5%) and VE was low (40%).

**Table 1. Estimated Statistical Significance of Differences in Influenza Incidence between Vaccinated and Unvaccinated Participants in a Cohort of 2,430 with Varying Vaccine Coverage, Incidence, and Hypothesized VE**

| **Vaccinated participant years (p-yrs)** | **Unvaccinated**  **p-yrs** | **Background incidence rate** | **Hypothesized VE** | **p-value *** |
| --- | --- | --- | --- | --- |
| 405  (40% vaccinated) | 607 | 5% | 40% | 0.13 |
| 405  (40% vaccinated) | 607 | 10% | 40% | 0.03 |
| 405  (40% vaccinated) | 607 | 5% | 50% | 0.05 |
| 405  (40% vaccinated) | 607 | 10% | 50% | 0.005 |
| 607  (60% vaccinated) | 405 | 5% | 40% | 0.11 |
| 607  (60% vaccinated) | 405 | 10% | 40% | 0.01 |
| 607  (60% vaccinated) | 405 | 5% | 50% | 0.04 |
| 607  (60% vaccinated) | 405 | 10% | 50% | 0.002 |
| ** p-value is for comparison of unadjusted influenza incidence rates in vaccinated vs unvaccinated* | | | | |

The full cohort provides the sample needs for estimating the frequency (or incidence within the study sample) of influenza illness. Table 2 presents the precision (plus and minus value of 95% confidence interval) associated with incidences of 5%, 10%, and 15%. Given the larger sample demands for the VE objectives, we expect to have ample statistical power for most objectives that involve estimating incidence or frequencies. However, estimating the frequency of influenza illness within specific HCP occupational or demographic subgroups may be challenging depending on the attack rate. For example, we expect to need at least 600 participants who complete surveillance activities in order to assess an incidence of 15% with a 95% confidence interval of 12-18%.

**Table 2. Plus and Minus Value for the Expected 95% Confidence Interval for Different Possible Incidences of Influenza-confirmed illness and Number of Participants**

|  | **N = 200** | **N = 300** | **N = 400** | **N = 500** | **N = 600** |
| --- | --- | --- | --- | --- | --- |
| **5% incidence** | +/- 3.0% | +/- 2.5% | +/- 2.1% | +/- 1.9% | +/- 1.7% |
| **10% incidence** | +/- 4.2% | +/- 3.4% | +/- 2.9% | +/- 2.6% | +/- 2.4% |
| **15% incidence** | +/- 4.9% | +/- 4.0% | +/- 3.5% | +/- 3.1% | +/- 2.9% |

**Data Analysis of Immunogenicity**

In sensitivity analyses, days between Time 1 and 2 sera collection and their association with Time 2 GMR will be examined.

To test the hypothesis that the outcome (serologic vaccine response or GMR) varies depending on the exposure (the number of prior IIV vaccinations), we will estimate an interaction term for time of sera draw (pre- and post-vaccination) by the number of prior vaccinations; after adjusting for main effects and covariates, a statistically significant interaction term (p < .1) indicated that vaccine response was significantly modified by prior IIV exposure.

Since baseline antibody titers are among the best predictors of serologic response [4], we will repeat the GMR models stratifying participants by those with low baseline titers (GMT <40) or high baseline titers (≥40) [1]. As an additional outcome measure and to aid the interpretation of the association between prior vaccinations and GMR, we will also report the percentage of participants with GMT of ≥40, a recognized immune marker associated with at least a 50% protection against influenza infection in populations [1], as well as a GMT of >100 which is associated with even higher clinical protection [2]. Given differences in baseline immunity, we will also report the percentage who achieved titers ≥40 and >100 post-vaccination, excluding those with preseason GMT ≥40. These percentages will be estimated using generalized linear models with the same covariates described for the mixed effects models and adjusted for baseline titers, as recommended [4], to minimize bias associated with prevaccination titers. We will use logistic regression, with the same covariates, to illustrate the magnitude of the effect of prior vaccination on the dichotomous elevated titer outcomes.

Vaccine exposure groups whose 95% confidence intervals (CI) (for GMT, GMR, or percentage with elevated titers) do not overlap will be considered statistically different. For other analyses, a p-value of < .05 will be considered statistically significant. Partial eta squared (η^2^) will be reported for mixed effect models to indicate the amount of variance in the outcome explained by the number of prior vaccinations after excluding variance explained by other covariates.

References

1. Gaglani M, Spencer S, Ball S, et al. Antibody response to influenza A(H1N1)pdm09 among healthcare personnel receiving trivalent inactivated vaccine: effect of prior monovalent inactivated vaccine. The Journal of infectious diseases **2014**; 209(11): 1705-14.

2. Coudeville L, Bailleux F, Riche B, Megas F, Andre P, Ecochard R. Relationship between haemagglutination-inhibiting antibody titres and clinical protection against influenza: development and application of a bayesian random-effects model. BMC Med Res Methodol **2010**; 10: 18.

3. Thompson MG, Gaglani MJ, Naleway A, et al. The expected emotional benefits of influenza vaccination strongly affect pre-season intentions and subsequent vaccination among healthcare personnel. Vaccine **2012**; 30(24): 3557-65.

4. Beyer WE, Palache AM, Luchters G, Nauta J, Osterhaus AD. Seroprotection rate, mean fold increase, seroconversion rate: which parameter adequately expresses seroresponse to influenza vaccination? Virus Res **2004**; 103(1-2): 125-32.
